# Coupling of *C*-nitro-*NH*-azoles with arylboronic acids. A route to *N-*aryl-*C*-nitroazoles

**DOI:** 10.3762/bjoc.9.173

**Published:** 2013-07-30

**Authors:** Marta K Kurpet, Aleksandra Dąbrowska, Małgorzata M Jarosz, Katarzyna Kajewska-Kania, Nikodem Kuźnik, Jerzy W Suwiński

**Affiliations:** 1 Department of Organic Chemistry, Bioorganic Chemistry and Biotechnology, Silesian University of Technology, Krzywoustego 4, Gliwice 44-100, Poland; 2 Centre of Polymer and Carbon Materials, Polish Academy of Sciences, M. Curie-Skłodowskiej 34, Zabrze 41-819, Poland

**Keywords:** arylboronic acids, *C*-nitroazoles, coupling, *N*-arylation, *N*-aryl-*C*-nitroazoles

## Abstract

A method for the synthesis of *N*-aryl-*C*-nitroazoles is presented. A coupling reaction between variously substituted arylboronic acids and 3(5)-nitro-1*H*-pyrazole catalyzed by copper salt has been carried out in methanol in the presence of sodium hydroxide to afford the desired *N*-aryl-*C*-nitroazoles in good yields. This synthetic route has also been successfully applied to obtain *N*-phenyl derivatives of 4-nitropyrazole, 2-nitroimidazole, 4(5)-nitroimidazole and 3-nitro-1,2,4-triazole.

## Introduction

The nitroazoles constitute a class of compounds with a broad spectrum of useful properties. They have found applications in agrochemicals as plant-growth regulators [1], herbicides or insecticides [2], in veterinary science [3], and as propellants and precursors of energetic materials [4]. Special attention has been paid to the development of their application in medicine where so far they are used as antiprotozoal, antifungal, and antibacterial drugs [5–6], as hypoxic cell sensitizers in radiation therapy of cancer [7], or as antiphlogistic drugs [8]. Many derivatives of *C*-nitroazoles, particularly *N*-alkylnitroimidazoles being the most often repeating subunit in biologically active compounds, suffer from mutagenic and carcinogenic properties [9]. Therefore the efforts of several research groups are focused on finding new compounds bearing *C*-nitroazole rings in their structure but exhibiting a less toxic effect.

Recent studies on biologically active derivatives of nitroazoles led to the synthesis and wide investigation of antibacterial and antiprotozoal 4-nitroimidazoles functionalized with an aryl substituent on the ring nitrogen atom. Many of these compounds exhibit a potent and selective anti-trypanosomal activity with relatively low mutagenic characteristics as well as genotoxic risk [10] and show promising antitubercular properties [11]. This opens an opportunity to look for new *N*-aryl-*C*-nitroazoles with the desired biological activity. Very recently, we reviewed the syntheses of *N*-phenyl-*C*-nitroazoles [12], and syntheses of their derivatives will be reviewed soon. The range of methods of *N*-aryl-*C*-nitroazoles synthesis includes nitration [13], ring closure [14–15], degenerated ANRORC reactions [16], transformation of other cyclic molecules [17], oxidation of aminoazoles [18], and introduction of an aryl substituent at the ring nitrogen atom in starting nitroazole [19–20]. Many of these methods suffer due to the inconvenient conditions of reactions, lack of selectivity, or low yields of the products. Therefore, there is a need for the development of a new, simple synthetic route, applicable to a wide range of *C*-nitro-*NH*-azoles.

Since 1998 when Chan and Lam [21–22] described a novel method for the formation of C–N bonds through the copper-catalyzed coupling of NH-containing substrates such as amines, amides, imides or heterocycles with boronic acids, the method has become a powerful synthetic route for *N*-arylation. Many investigations concerning the influence of the amount and kind of a catalyst, type of solvent, temperature, the presence of a base, and other reaction conditions, have carried out [23–27].

We were interested in developing an easy and effective method for the synthesis of *N*-aryl-*C-*nitroazoles that would allow us to obtain a broad range of the desired products bearing both chemically and electronically varying substituents on the benzene ring. The presence of the nitro group (a strong electron-withdrawing substituent) in the azole entails the adjustment of the conditions of *N*-arylation reaction.

Besides a very recently published approach involving *N*-arylation of nitroazoles with diaryliodonium salts [20], already described methods utilize arylboronic acids and substituted *C*-nitroazole coupling, applying the conditions described for the Chan–Lam reaction. This involves the use of chlorinated solvents, pyridine as base, and prolonged reaction times to obtain high yields of the product [28–29]. No detailed investigation on the influence of the conditions on the yield of the product has been presented.

## Results and Discussion

The group of products that we mainly describe is the 1-aryl derivatives of 3(5)-nitro-1*H*-pyrazole (**1a**). This is still a barely investigated group of compounds that can have attractive properties. Some recent reports describe them as new glucagon receptor antagonists [28] and compounds having insectidal properties [30]. They are a substructure of compounds being inhibitors of cannabinoid receptor 1 [31], they can be used as intermediate products, e.g., in the synthesis of compounds for inhibiting phosphate transport [32], and are described as being useful for the treatment of cognitive-deficit-associated psychiatric, neurodegenerative and neurological disorders [33].

**Scheme 1:**
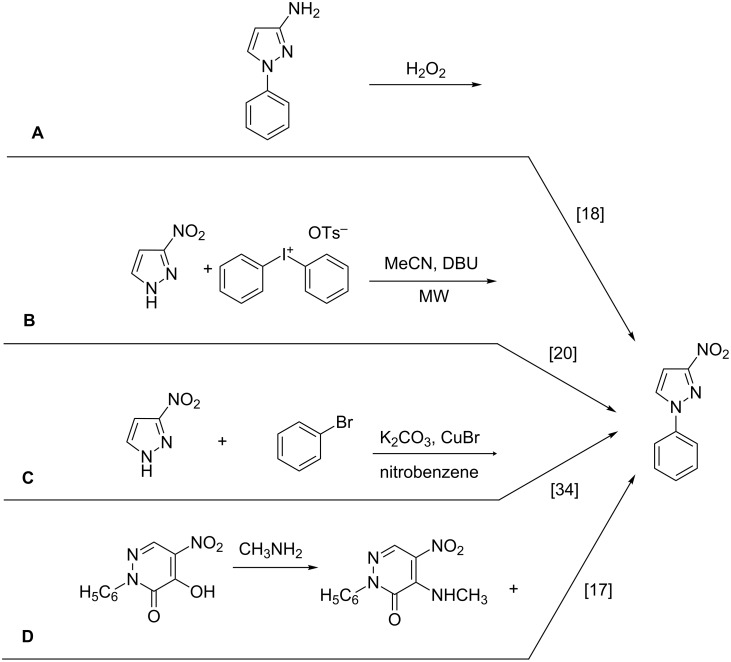
Methods of synthesis of 3-nitro-1-phenyl-1*H*-pyrazole (**3a**) described in the literature.

Only four papers describe the synthesis of 3-nitro-1-phenyl-1*H*-pyrazole. It may be a product of oxidation of 3-amino-1-phenyl-1*H*-pyrazole [18], a product of coupling of diaryliodonium salts with 3-nitro-1*H*-pyrazole [20], a product of Ullman phenylation of 3-nitro-1*H*-pyrazole [34], or a byproduct in the synthesis of 1-phenyl-4-amino-5-methylaminopyridazin-6-one [17] (Scheme 1).

Although these works describe 3-nitro-1-phenyl-1*H*-pyrazole (**3a**) as the product of the reaction, different physical properties are specified below.

Despite, we obtained 3-nitro-1-phenyl-1*H*-pyrazole (**3a**), for which the spectra determined in chloroform-*d* were in excellent agreement with those presented by Chertkov et al. for their product [20], the melting points differed from each other by almost 30 °C.

The melting point of the product synthesized by us is 98–99 °C, which is consistent with results obtained by Coburn [18], who oxidized 3-amino-1-phenyl-1*H*-pyrazole (Scheme 1, method A), while Chertkov et al. determined a value of 127 °C (Scheme 1, method B), which on the other hand fits with the data published by Predvoditeleva [17] (Scheme 1, method D).

The presence of tautomeric forms of 3(5)-nitro-1*H*-pyrazole (**1a**) and the discrepancy in the melting point of our product and the recently described one, led us to look carefully into its structure and to investigate the possibility of formation of other isomers.

The analysis of ^13^C NMR spectra of our products shows that there are characteristic signals, which can be assigned to carbon atoms in the 3-nitro-pyrazole rings: about 103–105 ppm for C^4-Py^, 129–132 ppm for C^5-Py^ and 156–157 ppm for C^3-Py^. These results are consistent with the analysis of regioisomers of 1-substituted-*C*-nitropyrazoles presented by Larina and Lopyrev in their review on nitroazoles [35]. Table 1 contains exemplified structures of 3-nitro-, 4-nitro- and 5-nitropyrazole derivatives and shows the differences in chemical shifts of carbon atoms in particular isomers. The examples do not include the 1-aryl substituent, which is present in our product, but general trends in chemical shifts can be observed. The C–NO_2_ signal in 5-nitropyrazole is shifted by about 10 ppm downfield in comparison to the C–NO_2_ signal in 3-nitropyrazole, what agrees with spectra recorded by us.

**Table 1:** ^13^C NMR chemical shifts (ppm) of *C*-nitropyrazoles [35].

Substituents in nitropyrazole ring				^13^C NMR chemical shifts (ppm) for nitropyrazoles			
R^1^	R^3^	R^4^	R^5^	C^3^	C^4^	C^5^	solvent

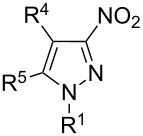							
H	–	H	H	155.70	103.24	132.80	CD_3_OD
CH_3_	–	H	H	154.90	102.70	134.50	DMSO-*d*_6_
NH_2_	–	H	H	152.96	102.17	132.80	DMSO-*d*_6_

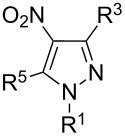							
H	H	–	H	132.41	136.00	132.44	DMSO-*d*_6_
CH_3_	H	–	H	135.00	134.90	130.60	DMSO-*d*_6_
NH_2_	H	–	H	132.96	133.33	128.32	DMSO-*d*_6_

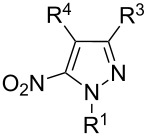							
CH_3_	H	H	–	137.60	106.30	145.80	DMSO-*d*_6_
NH_2_	H	H	–	133.60	104.72	142.26	DMSO-*d*_6_

**Figure 1:**
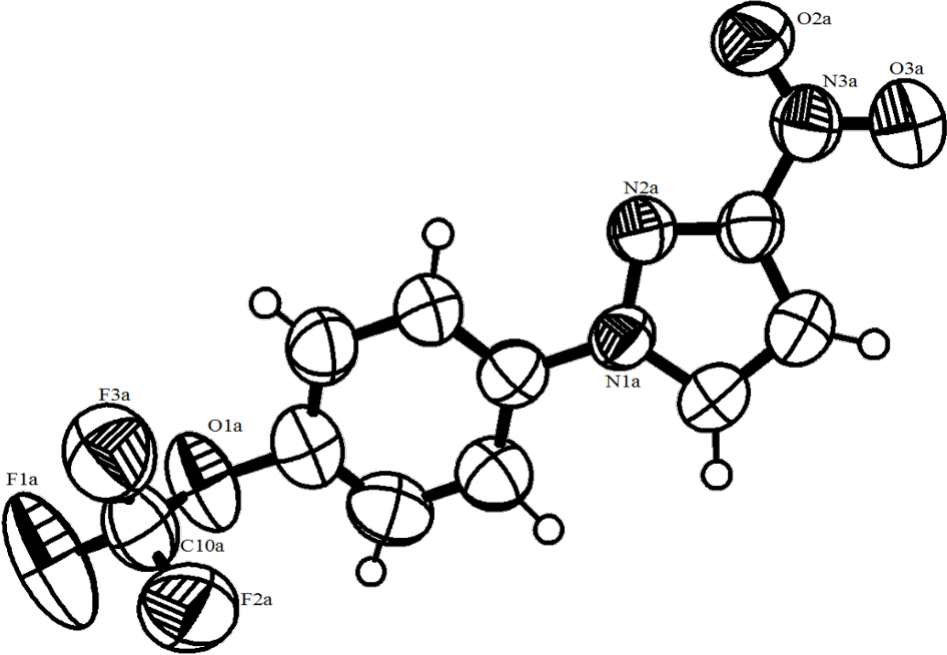
X-ray structure of 3-nitro-1-[4-(trifluoromethoxy)phenyl]-3-nitro-1*H*-pyrazole (**3k**) with 60% probability ellipsoids.

Additionally, in order to confirm the structure we used X-ray analysis for one of our products. The analyzed crystal of 3-nitro-1-[4-(trifluoromethoxy)phenyl]-3-nitro-1*H*-pyrazole (**3k**) was a monocrystal recrystallized from diethyl ether (Figure 1). It forms a monoclinic unit cell with two symmetry-related pairs of molecules. The molecule is relatively flat, with the benzene ring slightly twisted out from the pyrazole plane 19.60° (27), while the nitro-substituent is almost coplanar with pyrazole root 8.06° (90). Such a structure enables efficient overlapping of π orbitals resulting in high conjugation.

Based on the agreement of our NMR spectra with those presented and thoroughly analyzed by Chertkov et al., as well as on our X-ray analysis, the substitution of the nitro-group in the 3-position of the 1*H*-pyrazole moiety was confirmed.

**Scheme 2:**
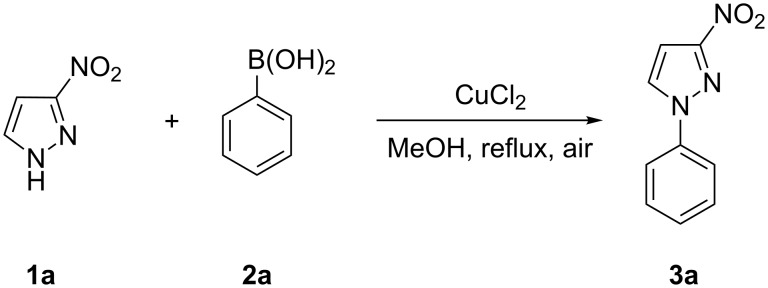
Cross coupling of 3-nitro-1*H*-pyrazole (**1a**) with phenylboronic acid (**2a**).

Our screening of the conditions involved the factors influencing the chemical yield of reaction, including the solvent/base system, the type of catalyst, and the stoichiometry of the reagents. 3-Nitro-1*H*-pyrazole (**1a**) and phenylboronic acid (**2a**) were used as model substrates to optimize the reaction conditions (Scheme 2).

At the very beginning we decided to perform several experiments that would allow us to establish the necessity of the presence of a catalyst, air and a base in the reaction system (Table 2). The initial conditions of the reaction were based on those published by Lan et al. [24] for coupling imidazole with phenylboronic acid. Most reports on the Chan–Lam coupling reaction underline the demand of air introduction into the reaction mixture to provide high yields of the products [22,24,36–37]. The plausible mechanism of this catalytic reaction was proposed by Evans [38] and described for *N*-nucleophiles by Collman [39]. It involves several steps: transmetallation of boronic acid with a catalyst, coordination of the azole molecule to the Cu(II) species followed by oxidation of copper(II) into copper(III) in the presence of oxygen, and then reductive elimination releasing the product and Cu(I) complex. A regeneration of the catalyst takes place in the presence of oxygen reproducing the Cu(II) cation.

**Table 2:** The influence of the presence of air, a base and a catalyst on the yield of 3-nitro-1-phenyl-1*H*-pyrazole (**3a**).^a^

Entry	Catalyst	Base	Air	Yield^b^

1	CuI	no	no^e^	0%
2^c^	CuI	no	+	trace
3^d^	CuI	NaOH	no^e^	trace
4^c,d^	CuI	NaOH	+	64%
5^d^	CuCl_2_	NaOH	no^e^	56%
6^c,d^	CuCl_2_	NaOH	+	69%
7^c,d^	–	NaOH	+	0%

^a^Conditions: 2 mmol of **2a**, 2.4 mmol of **1a**, 10 mol % of catalyst, methanol, reflux. The progress of the reaction was controlled by TLC. ^b^Isolated yields. ^c^Air was bubbled through solution. ^d^In the presence of 2.4 mmol of NaOH. ^e^No bubbling of air through solution.

In the case of the application of Cu(I) salts, for which our preliminary studies were carried out, the presence of air proved to be obligatory. Attempts to perform the reaction without air resulted in no or only trace amounts of product being detected on the TLC plate (Table 2, entries 1 and 3). Our further experiments carried out in the presence of Cu(II) salt revealed that the air does not seem to be necessarily introduced for the reaction to take place (Table 2, entry 5). However, bubbling air through the reaction mixture improved the yield (Table 2, entry 6), indicating its possible impact on the catalytic cycle. These results are in agreement with those obtained by Strijdonck [40] who carried out copper-catalyzed coupling under anaerobic conditions. The mechanism of this reaction is still a matter of research. The reaction does not take place without a base (Table 2, entry 2) or in the absence of copper catalyst (Table 2, entry 7).

A parameter influencing chemical yields to a greater extent was the applied solvent/base system. Sodium hydroxide (p*K*_a_ = 15.7) [41] and triethylamine (Et_3_N) (p*K*_a_ = 10.9) [42] in dichloromethane, acetonitrile, dimethylformamide, tetrahydrofuran and methanol were investigated. Dichloromethane, being the most often reported solvent for coupling [21–23,36], turned out to be the least appropriate in the case of *C*-nitroazoles. Yields of the product obtained were then 3% and 8% in the presence of Et_3_N and NaOH, respectively (Table 3, entries 1,2). Even prolonged time did not improve the yield (Table 3, entry 3). Better results were obtained for more polar solvents (acetonitrile, dimethylformamide, tetrahydrofuran, methanol) independently on a base (Table 3, entries 4–11). The most efficient combinations were dimethylformamide/sodium hydroxide and methanol/sodium hydroxide. Methanol was the solvent of choice due to higher yields and convenience of workup and purification of the product.

**Table 3:** Influence of the solvent/base system on the yield of **3a**.^a^

Entry	Solvent	Base	Time [h]	Yield^b^

1	CH_2_Cl_2_	NaOH	11	8 %
2	CH_2_Cl_2_	Et_3_N	10	3%
3	CH_2_Cl_2_	NaOH	20	8%
4	CH_3_CN	NaOH	11	48%
5	CH_3_CN	Et_3_N	11	40%
6	DMF	NaOH	9	56%
7	DMF	Et_3_N	11	40%
8	THF	NaOH	10	48%
9	THF	Et_3_N	11	37%
10	CH_3_OH	NaOH	10	64%
11	CH_3_OH	Et_3_N	11	26%
12	CH_3_OH	K_2_CO_3_	13	25%
13	CH_3_OH	DBU	12	63%

^a^Conditions: 2 mmol of **2a**, 2.4 mmol of **1a**, 10 mol % of CuI, 2.4 mmol of base, methanol, reflux, air bubbled through solution. The progress of the reaction was controlled by TLC. ^b^Isolated yields.

In order to complete the research on the solvent/base system, the coupling reaction was carried out in methanol in the presence of other bases such as potassium carbonate (p*K*_a_ = 10.33) [43] and 1,8-diazabicyclo[5.4.0]undec-7-ene (DBU, p*K*_a_ = 12) [44] (Table 3, entries 12,13). Although the p*K*_a_ values were determined in water, they are usually in good agreement in other polar solvents scales [45]. Not surprisingly, the differences in p*K*_a_ values of the bases are reflected in the results obtained. Very strong bases (DBU, NaOH) are needed for the reaction to take place. Although the reaction with DBU yielded a similar amount of product as NaOH, the latter was chosen as being of lower toxicity, easier to process and environmentally friendly.

Next the catalytic activity of the copper salts was screened. Most known reports concerning *N*-arylation focus on Cu(OAc)_2_ salt [21–22], complexes of Cu(II) with different ligands [23,36], and heterocyclic copper-based catalysts [25,37]. Basically, easily available simple copper(I) and copper(II) compounds, mostly salts, were investigated. The yields obtained for Cu(I) salts (Table 4, entries 1–3) were slightly lower than those achieved for Cu(II) salts (Table 4, entries 5–8). However, the differences are small, and it can be assumed that under the reaction conditions applied the choice between Cu(I) and Cu(II) is not crucial, considering the yield of 3-nitro-1-phenyl-1*H*-pyrazole. Also the effect of the counterion seems to be insignificant. Among the investigated compounds only CuO was ineffective as a catalyst and gave only trace amounts of the product as detected on the TLC plate. This implies the need to consider another factor. The use of Cu(I) species obligates the introduction of air into the reaction mixture during the reaction. As our previous experiments confirmed, it is not required for Cu(II) salts, although it provides higher yields of the product.

**Table 4:** The influence of copper catalyst on the yield of **3a**.^a^

Entry	Catalyst	Yield^b^

1	CuI	63%
2	CuCl	66%
3	CuBr	58%
4	CuO	trace
5	CuCl_2_	69%
6	Cu(OAc)_2_·H_2_O	66%
7	CuSO_4_	66%
8	Cu(acac)_2_	53%

^a^Conditions: 2 mmol of 2a, 2.4 mmol of 1a, 10% mol of a catalyst, 2.4 mmol of NaOH, methanol, reflux, 10 h, air bubbled through solution. The progress of the reaction was controlled by TLC. ^b^Isolated yields.

It turned out that the ratio of reagents has an important influence on the chemical yield of 3-nitro-1-phenyl-1*H*-pyrazole. A ratio of arylboronic acid to 3-nitro-1*H*-pyrazole to the base of 2.6:1.6:1.6 seems to be optimal. This gave the highest (up to 82%) yield of the product. A series of experiments, when either an excess of azole or a base over other reagents was used, resulted in decreased yields. Nevertheless, the base should be used in stoichiometric proportion to the azole. Both increasing and decreasing the proportion led to lower yield of the product.

**Scheme 3:**
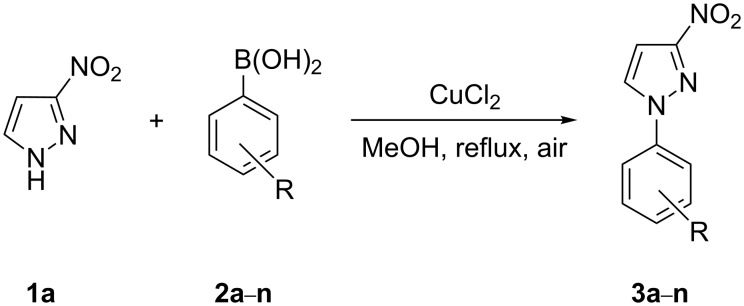
Cross coupling of **1a** with arylboronic acids **2a**–**n**.

With an efficient *N*-arylating system for phenylboronic acid, we expanded the scope of this reaction by exploring a variety of boronic acids with different substitution patterns. The investigated compounds brought such substituents as F, Cl, CH_3_, OCH_3_, OCF_3_, and NO_2_ (Scheme 3). The results show that an electron-donating character of a substituent (Table 5, entries 7–10) allows higher yields of the products to be obtained than with electron-withdrawing groups (Table 5, entries 2–6 and entries 11–14).

**Table 5:** Coupling of **1a** with arylboronic acids **2a–n**.^a^

Entry	Boronic acid	Product	Time [h]	Yield^b^

1	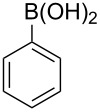 **2a**	**3a**	18	82%
2	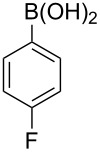 **2b**	**3b**	11	64%
3	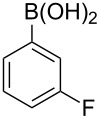 **2c**	**3c**	11	58%
4	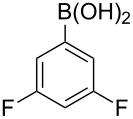 **2d**	**3d**	25	50%
5	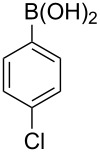 **2e**	**3e**	16	67%
6	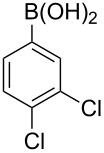 **2f**	**3f**	8	44%
7	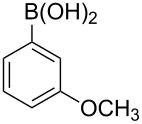 **2g**	**3g**	8	74%
8	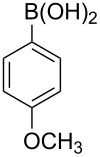 **2h**	**3h**	14	86%
9	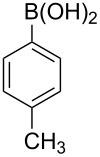 **2i**	**3i**	20	80%
10	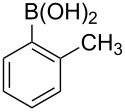 **2j**	**3j**	13	86%
11	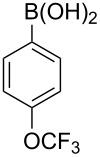 **2k**	**3k**	15	66%
12	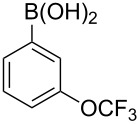 **2l**	**3l**	15	64%
13	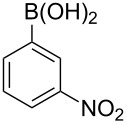 **2m**	**3m**	31	62%
14	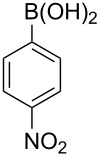 **2n**	**3n**	30	65%

^a^Conditions: 2.6 mmol of arylboronic acid **2a–n**, 1.6 mmol of **1a**, 10 mol % of a catalyst, 1.6 mmol of NaOH, methanol, reflux, air bubbled through the solution. The progress of the reaction was monitored by TLC. ^b^Isolated yields.

The yields also vary with the location of the substituent on the benzene ring. *Ortho*- and *meta*-substituted phenylboronic acids gave in most cases lower yields in comparison to *para*-substituted ones (Table 5, entries 2,3,5–8,11–14). This might be a result of steric hindrance around the reaction center in the catalytic cycle.

**Scheme 4:**
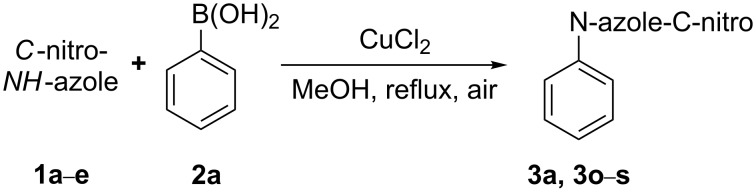
Cross coupling of *C*-nitro-*NH*-azoles **1a**–**d** with phenylboronic acid (**2a**).

Applicability of the synthetic procedure to the preparation of various *N*-aryl-*C*-nitroazoles was also investigated. For this purpose the cross coupling of phenylboronic acid with a series of *C*-nitro-*NH-*azoles such as: 3(5)-nitropyrazole (**1a**) (p*K*_a_ = 9.81) [46], 4-nitropyrazole (**1b**) (p*K*_a_ = 9.67) [46], 4(5)-nitroimidazole (**1c**) (p*K*_a_ = 8.93) [47], 3-nitro-1,2,4-triazole (**1d**) (p*K*_a_ = 6.05) [48] and 2-nitroimidazole (**1e**) (p*K*_a_ = 7.15 in CH_3_OH/H_2_O 1:1) [49] (Scheme 4, Table 6) was carried out. The results show that this method allows *N*-aryl derivatives of *C*-nitroazoles to be obtained within a wide p*K*_a_ range.

**Table 6:** Cross coupling of *C*-nitro-*NH*-azoles **1a–e** with 2a.^a^

Entry	*C*-nitro-*NH*-azole	Product	Time [h]	Yield^b^

1	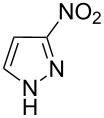 **1a**	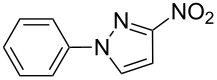 **3a**	18	82%
2	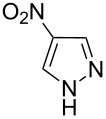 **1b**	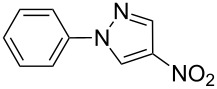 **3o**	15	53%
3	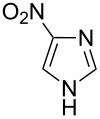 **1c**	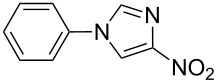 **3p**	18	86%
4	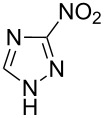 **1d**	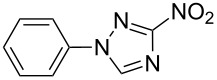 **3r**	16	50%
5	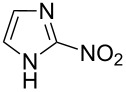 **1e**	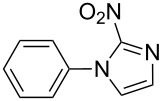 **3s**	9	40%

^a^Conditions: 2.6 mmol of **2a**, 1.6 mmol of *C*-nitroazole **1a**–**e**, 10 mol % of a catalyst, 1.6 mmol of NaOH, methanol, reflux, air bubbled through the solution. The progress of the reaction was controlled by TLC. ^b^Isolated yields.

Comparing our results presented here with those reported very recently by V. A. Chertkov et al. [20], it appears that both methods are regioselective and lead to syntheses of *N*-aryl-*C*-nitroazoles in moderate to high yields. Arylboronic acids are more easily available than diaryliodonium salts, and thus the scope of our approach seems to be wider. The much milder reaction conditions presented in this paper make this route more valuable.

## Conclusion

In conclusion, we have developed an efficient and simple method for the cross coupling of arylboronic acids with *C*-nitro-*NH*-azoles in the presence of a catalytic amount of simple copper salts. The reaction takes place in a protic solvent containing a base, both of which are necessary for providing good yields of the products. The method represents an important supplement to the synthetic methodologies for the preparation of *N*-aryl-*C*-nitroazoles and can be successfully applied to the synthesis of a series of diverse *C*-nitroazoles functionalized with an aryl substituent on a ring nitrogen atom.

## Supporting Information

File 1 Experimental procedures and characterization of products.

File 2 Crystallographic information file (structure of 3-nitro-1-[4-(trifluoromethoxy)phenyl]-3-nitro-1*H*-pyrazole).
