# Supplementary material for: Coupling of C-nitro-NH-azoles with arylboronic acids. A route to N-aryl-C-nitroazoles
Source: Beilstein J Org Chem. 2013 Jul 30;9:1517–25. doi: 10.3762/bjoc.9.173 (PMC3740504; doi:10.3762/bjoc.9.173)
Supplement: File 1 — Experimental procedures and characterization of products. [file Beilstein_J_Org_Chem-09-1517-s001.pdf]

Supporting Information

for

Coupling of *C*-nitro-*NH*-azoles with arylboronic acids. A route to *N*-aryl-*C*-nitroazoles

Marta K. Kurpet[1]*, Aleksandra Dąbrowska[1], Małgorzata M. Jarosz[1], Katarzyna Kajewska-Kania[1], Nikodem Kuźnik[1], Jerzy W. Suwiński[2]

Address: [1]Department of Organic Chemistry, Bioorganic Chemistry and Biotechnology, Silesian University of Technology, Krzywoustego 4, Gliwice 44-100, Poland and [2]Centre of Polymer and Carbon Materials, Polish Academy of Sciences, M. Curie-Skłodowskiej 34, Zabrze 41-819, Poland

E-mail: Marta Kurpet - marta.kurpet@polsl.pl

* Corresponding author

Experimental procedures and characterization of products

Experimental part:

The melting points (not corrected) were determined with a Boetius HMK apparatus or with an open capillary. ^{1}H and ^{13}C spectra were recorded on a Varian XL-300 (300 MHz for ^{1}H, 75.5 MHz for ^{13}C), Agilent 400MHz (400 MHz for ^{1}H, 100 MHz for ^{13}C) on a Varian 600 (600 MHz for ^{1}H, 150 MHz for ^{13}C) in DMSO-d_6 or chloroform-*d* and with tetramethylsilane as the internal reference. The chemical shifts (δ) are reported in parts per million and the coupling constants (*J*) in hertz. Elemental analyses (EA) were performed using a 2400 Series

II CHNS/O Elemental Analyzer. Electrospray-ionization mass spectrometry (ESIMS) was performed on a 4000 QTrap (Applied Biosystems/MDS Sciex) mass spectrometer.

4-Methoxy-, 4-trifluoromethoxy-, 2-methyl-phenylboronic acids were purchased from Maybridge, 3-nitro-1,2,4-triazole was purchased from Aldrich, and other arylboronic acids and 2-nitroimidazole were supplied by Acros Organics. TLC 60F254 plates and silica gel 60 (0.040–0.063 mm) were purchased from Merck. All reagents were used without further purification. 3(5)-Nitropyrazole was obtained in our laboratory by a modified procedure of the thermal rearrangement of *N*-nitropyrazole following nitration of pyrazoles [1]. 4-Nitropyrazole was obtained according to the known procedure [2].

X-ray single-crystal measurement: measurements of the diffraction intensities were performed on a KUMA KM4 four-circle diffractometer, MoK_{α} radiation, $\omega/2\Theta$ scan mode, Θ range 2.12–25.06°. Crystallographic data for **3k** were deposited with the Cambridge Crystallographic Data Centre with publication number: CCDC 935159. A complete listing of the atomic coordinates of x, y, and z can be obtained free of charge, on request, from the Director, Cambridge Crystallographic Data Centre, 12 Union Road, Cambridge CB2 1EZ, UK [fax: (+int) 44-1223 336 033; e-mail: deposit@ccdc.cam.ac.uk], upon quoting the depository numbers, names of the authors, and the journal citation.

Synthesis of 3(5)-nitropyrazole (1a)

Fuming nitric acid (3.3 mL) was added dropwise to a stirred solution of pyrazole (4.5 g, 66 mmol) in glacial acetic acid (14.1 mL) that had been cooled to −10 °C using an ice-salt bath. A voluminous precipitate was formed. Acetic anhydride (9.45 mL) was added dropwise and the resultant mixture was stirred at ambient temperature for 1.5 h. The mixture was poured onto ice and was neutralized with potassium carbonate. The precipitate was isolated by filtration. The yield of the obtained white solid of *N*-nitropyrazole is 78%. Mp 91-92 °C (Lit [1]: 91-92 °C);

^{1}H NMR (300 MHz, DMSO): δ (ppm) = 6.71-6.73 (m, 1H, H^4), 7.90 (d, *J* = 0.9 Hz, 1H, H^3), 8.81 (dd, *J* = 2.7 Hz, *J* = 0.9 Hz, 1H, H^5); ^{13}C NMR (75 MHz, DMSO): δ (ppm) = 110.1 (C^4), 127.2 (C^3), 141.9 (C^5).

N-Nitropyrazole (2 g, 18 mmol) was heated at 145 °C for 10 h in a round-bottom flask. Then the product was purified by silica column chromatography using 5:95 v/v methanol/chloroform as eluent to afford 56% of 3(5)-nitropyrazole as a white solid. Mp 174.5–176 °C (Lit [1]: 174–175 °C); ^{1}H NMR (300 MHz, DMSO): δ (ppm) = 7.02 (d, *J* =

2.7 Hz, 1H, H^{4}), 8.02 (d, *J* = 2.7 Hz, 1H, $H^{5(3)}$), 13.92 (bs, 1H, NH); ^{13}C NMR (75 MHz, DMSO): δ (ppm) = 101.3, 131.3, 156.37.

4-Nitropyrazole (1b)

Pyrazole (8.5 g) and cold concentrated nitric acid (18 cm^3, *d* = 1.4 g/mL) were added to concentrated sulfuric acid (15 cm^3) that had been cooled to 0 °C using an ice–salt bath. The reaction mixture was heated under reflux for 3 h. The reaction mixture was cooled to room temperature, and additional nitrating mixture (6 cm^3 of concentrated sulfuric acid and 6 cm^3 of nitric acid) was added dropwise. The mixture was heated under reflux for a further 3 h, cooled and left to stand overnight. The obtained solution was poured onto ice (80 g), and the precipitate was filtered under reduced pressure, washed with cold water and cold ethanol, and crystallized from toluene. 4-Nitropyrazole (56 % yield) was obtained as a white solid. Mp 158–159.5 °C (Lit. [3] 160–161 °C)

General procedure for the cross-coupling reaction:

A mixture of 1.6 mmol of *C*-nitro-*NH*-azole **(1a–e)**, 2.6 mmol of arylboronic acid **(2a-n)**, 1.6 mmol of sodium hydroxide, 0.2 mmol of $CuCl_2$ and methanol (15 mL) was refluxed while air was bubbled through the reaction mixture. After completion of the reaction, determined on the basis of TLC analysis, the solvent was removed under reduced pressure using a rotary evaporator. The obtained crude product was purified by silica gel column chromatography with 5:95 v/v MeOH/$CHCl_3$ as an eluent to give corresponding *N*-aryl-*C*-nitroazole. The product was crystallized from methanol/water.

3-Nitro-1-phenyl-1*H*-pyrazole (3a)

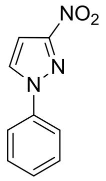

White solid, yield 82%, mp 98–99 °C (lit. 98–99 °C [4], 126–128 °C [5])

^{1}H NMR (600 MHz, DMSO): δ (ppm) = 7.36 (d, *J* = 2.8 Hz, 1H, $H^{4\text{-}Py}$), 7.49 (m, 1H, H^{4}), 7.61 (m, 2H, $H^{3,5}$), 7.94 (dd, *J* = 9.0 Hz, *J* = 1.0 Hz, 2H, $H^{2,6}$), 8.79 (d, *J* = 2.8 Hz, 1H, $H^{5\text{-}Py}$); ^{13}C NMR (150 MHz, DMSO): δ (ppm) = 104.66 ($C^{4\text{-}Py}$), 119.60 ($C^{2,6}$), 128.52 (C^{4}), 129.90 ($C^{3,5}$), 131.86 ($C^{5\text{-}Py}$), 138.52 (C^{1}), 156.53 ($C^{3\text{-}Py}$).

^{1}H NMR (400 MHz, $CDCl_3$): δ (ppm) = 7.09 (d, *J* = 2.8 Hz, 1H, $H^{4\text{-}Py}$), 7.41-7.45 (m, 1H, H^{4}), 7.49-7.54 (m, 2H, $H^{3,5}$), 7.73-7.76 (m, 2H, $H^{2,6}$), 7.98 (d, J^3=2.8 Hz, 1H, $H^{5\text{-}Py}$); ^{13}C NMR (100 MHz, $CDCl_3$): δ (ppm) = 104.33 ($C^{4\text{-}Py}$), 120.05 ($C^{2,6}$), 128.66 (C^{4}), 129.51 ($C^{5\text{-}Py}$), 129.76 ($C^{3,5}$), 138.85 (C^{1}), 157.04 ($C^{3\text{-}Py}$); EA for $C_9H_7N_3O_2$, calc: C 57.14, H 3.73, N 22,21; found: C 57.16, H 3.58, N 22.18; ESIMS: *m*/*z*: 190.2 (M + H)

1-(4-Fluorophenyl)-3-nitro-1*H*-pyrazole (3b)

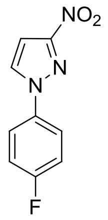

Pale peach solid, yield 64%; mp 148–149 °C; ^{1}H NMR (400 MHz, DMSO): δ (ppm) = 7.35 (d, *J* = 2.8 Hz, 1H, $H^{4\text{-}Py}$), 7.42-7.48 (m, 2H, $H^{3,5}$), 7.95-7.99 (m, 2H, $H^{2,6}$), 8.77 (d, J^3=2.8 Hz, 1H, $H^{5\text{-}Py}$); ^{13}C NMR (100 MHz, DMSO): δ (ppm) = 104.57 (s, $C^{4\text{-}Py}$), 116.61 (d, J^2=22.8 Hz, $C^{3,5}$), 121.89 (d, J^3=9.1 Hz, $C^{2,6}$), 132.02 (s, $C^{5\text{-}Py}$), 135.00 (d, J^4=3.0 Hz, C^{1}), 156.44 (s, $C^{3\text{-}Py}$), 161.47 (d, J^1=245.1 Hz, C^{4}); EA for $C_9H_6FN_3O_2$, calc: C 52.18, H 2.92, N 20.28, found: C 52.43, H 3.09, N 20.30; ESIMS: *m*/*z*: 208.0 (M + H)

1-(3-Fluorophenyl)-3-nitro-1*H*-pyrazole (3c)

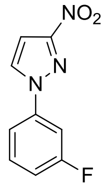

White solid, yield 58%, mp 108–110 °C; ^{1}H NMR (400 MHz, DMSO): δ (ppm) = 7.11 (d, *J* = 2.8 Hz, 1H, $H^{4\text{-}Py}$), 7.14-7.17 (m, 1H, H^{Ar}), 7.47-7.55 (m, 3H, H^{Ar}), 8.00 (d, *J* = 2.8 Hz, 1H, $H^{5\text{-}Py}$); ^{13}C NMR (100 MHz, DMSO): δ (ppm) = 104.80 (s, $C^{4\text{-}Py}$), 107.04 (d, J^2=26.5 Hz, C^{2}), 115.15 (d, J^2=21.3 Hz, C^{4}), 115.47 (d, J^4=3.1 Hz, C^{6}), 131. 78 (d, J^3=9.1 Hz, C^{5}), 132.23 (s, $C^{5\text{-}Py}$), 139.75 (d, J^4=10.6 Hz, C^{1}), 156.61 (s, $C^{3\text{-}Py}$), 162.45 (d, J^1=243.5 Hz, C^{3}); EA: calc. for $C_9H_6FN_3O_2$,: C 52.18, H 2.92, N 20.28; found: C 52.36, H 2.72, N 20.42; ESIMS: *m*/*z*: 208.1 (M+H)

1-(3,5-Difluorophenyl)-3-nitro-1*H*-pyrazole (3d)

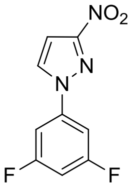

Light yellow solid, yield 58%, mp 132–134 °C; ^{1}H NMR (600 MHz, DMSO): δ (ppm) = 7.38-7.42 (m, 2H, H^4, $H^{4\text{-}Py}$), 7.73-7.76 (m, 2H, $H^{2,6}$), 8.85 (d, J^3=2.4 Hz, 1H, $H^{5\text{-}Py}$); ^{13}C NMR (150 MHz, DMSO): δ (ppm) = 103.78-104.35 (m, $C^{2,6}$, C^4), 105.52 (s, $C^{4\text{-}Py}$), 132.99 (s, $C^{5\text{-}Py}$), 140.79 (t, J = 12.6 Hz, C^1), 157.22 (s, $C^{3\text{-}Py}$), 163.34 (dd, J = 246.15 Hz, J = 15 Hz, ($C^{3,5}$); EA calc. for $C_9H_5F_2N_3O_2$: C 48.01, H 2.24, N 18.66; found: C 48.30, H 2.32, N 18.52

1-(4-Chlorophenyl)-3-nitro-1*H*-pyrazole (3e)

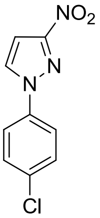

Light yellow, yield 67%**,** mp 113–115 °C; ^{1}H NMR (400 MHz, DMSO): δ (ppm) = 7.36 (d, J^3=2.8 Hz, 1H, $H^{4\text{-}Py}$), 7.96 (dd, J^3=6.4 Hz, J^3=1.6 Hz, 2H, $H^{3,5}$), 7.61 (d, J^3=8.8 Hz, 2H, $H^{2,6}$), 8.81 (d, J^3=2.8 Hz, 1H, $H^{5\text{-}Py}$); ^{13}C NMR (100 MHz, DMSO): δ (ppm) = 104.70 ($C^{4\text{-}Py}$), 121.13, 129.68, 131.92, 132.70, 137.21, 156.52($C^{3\text{-}Py}$); EA calc. for $C_9H_6ClN_3O_2$: C 48.34, H 2.70, N 18.79; found: C 48.50, H 2.52, N 18.92; ESIMS: *m*/*z*: 245.9 (M + Na)

1-(3,4-Dichlorophenyl)-3-nitro-1*H*-pyrazole (3f)

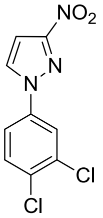

Light yellow, yield 44%**,** mp 173–174 °C; ^{1}H NMR (400 MHz, DMSO): δ (ppm) = 7.38 (d, J^{3}=2.8 Hz, 1H, H^{4-Py}), 7.86 (d, J^{3}=8.8 Hz, 1H, H^{5}), 7.95 (dd, J^{4}=2.8 Hz, J^{3}=8.8 Hz, 1H, H^{6}), 8.25 (d, J^{3}=2.4 Hz, 1H, H^{2Ar}), 8.87 (d, J^{3}=2.8 Hz, 1H, H^{5-Py}); ^{13}C NMR (100 MHz, DMSO): δ (ppm) = 104.94 (C^{4-Py}), 119.54, 121.17, 130.76, 130.85, 131.68, 132.37, 137.95, 156.71(C^{3-Py}); EA: calc for $C_9H_5Cl_2N_3O_2$: C 41.89, H 1.95, N 16.28; found: C 41.71, H 1.75, N 16.40; ESIMS: *m*/*z*: 280.1 (M + Na)

1-(3-Methoxyphenyl)-3-nitro-1*H*-pyrazole (3g)

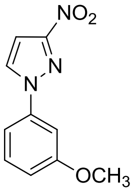

Peach solid, yield 74%, mp 113 °C; ^{1}H NMR (400 MHz, DMSO): δ (ppm) = 3.87 (s, 3H, $-OCH_3$), 7.05-7.06 (m, 1H, H^{5}), 7.35 (d, J^{3}=2.8 Hz, 1H, H^{4-Py}), 7.47-7.50 (m, 3H, $H^{2,4,6}$), 8.81 (d, J^{3}=2.8 Hz, 1H, H^{5-Py}); ^{13}C NMR (100 MHz, DMSO): δ (ppm) = 55.58, 104.51 (C^{4-Py}), 105.18, 111.54, 114.13, 130.75, 131.92, 139.53, 156.36(C^{3-Py}), 160.19 (C^{3}); EA, calc. for $C_{10}H_9N_3O_3$: C 54.79, H 4.14, N 19.17; found: C 54.97, H 3.89, N 19.26; ESIMS: *m*/*z*: 220.2 (M + H)

1-(4-Methoxyphenyl)-3-nitro-1*H*-pyrazole (3h)

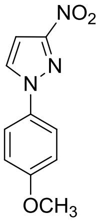

Yellow solid, yield 86%, mp 108–109 °C; ^{1}H NMR (400 MHz, DMSO): δ (ppm) = 3.84 (s, 3H, -OCH_3), 7.13 (m, 2H, $H^{3,5}$), 7.32 (d, J^3=2.4 Hz, 1H, $H^{4\text{-}Py}$), 7.84 (m, 2H, $H^{2,6}$), 8.68 (d, J^3=2.4 Hz, 1H, $H^{5\text{-}Py}$); ^{13}C NMR (100 MHz, DMSO): δ (ppm) = 55.52 (-OCH_3), 104.34 ($C^{4\text{-}Py}$), 114.79, 121.16, 131.49, 131.92, 156.06 ($C^{3\text{-}Py}$), 159.12 (C^4); EA calc for $C_{10}H_9N_3O_3$: C 54.79, H 4.14, N 19.17 ; found: C 54.96, H 4.09, N 18.79; ESIMS: *m*/*z*: 220.3 (M + H)

1-(4-Methylphenyl)-3-nitro-1*H*-pyrazole (3i)

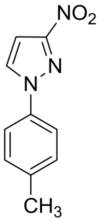

Light yellow solid, yield 80%**,** mp 89–91 °C; ^{1}H NMR (400 MHz, $CDCl_3$): δ (ppm) = 2.41 (s, 3H, -CH_3), 7.06 (d, J^3=2.8 Hz, 1H, $H^{4\text{-}Py}$), 7.29 (d, J^3=8.8 Hz, 2H, $H^{3,5}$), 7.61 (d, J^3=8.8 Hz, 2H, $H^{2,6}$), 7.94 (d, J^3=2.8 Hz, 1H, $H^{5\text{-}Py}$); ^{13}C NMR (100 MHz, $CDCl_3$): δ (ppm) = 21.66 (-CH_3), 104.84 ($C^{4\text{-}Py}$), 120.57, 130.14, 130.90, 137.50, 139.50, 156.83($C^{3\text{-}Py}$); EA calc. for $C_{10}H_9N_3O_2$: C 59.11, H 4.46, N 20.68, found: C 59.31, H 4.34, N 20.32; ESIMS: *m*/*z*: 204.0 (M + H)

1-(2-Methylphenyl)-3-nitro-1*H*-pyrazole (3j)

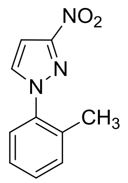

Salmon solid, yield 86%, mp 85–86 °C; ^{1}H NMR (400 MHz, CDCl$_3$): δ (ppm) = 2.25 (s, 3H, -CH$_3$), 7.07 (d, J^3=2.4 Hz, 1H, H$^{4\text{-Py}}$), 7.28-7.43 (m, 4H, H^{Ar}), 7.68 (d, J^3=2.4 Hz, 1H, H$^{5\text{-Py}}$); ^{13}C NMR (100 MHz, CDCl$_3$): δ (ppm) = 18.07 (-CH$_3$), 103.50 (C$^{4\text{-}Py}$), 126.41, 127.21, 130.25, 131.86, 133.85, 134.03, 138.89, 157.05 (C$^{3\text{-Py}}$); EA calc. for $C_{10}H_9N_3O_2$: C 59.11, H 4.46, N 20.68; found: C 58.96, H 4.64, N 20.34; ESIMS: *m*/*z*: 226.1 (M + Na)

3-Nitro-1-[4-(trifluoromethoxy)phenyl]-3-nitro-1*H*-pyrazole (3k)

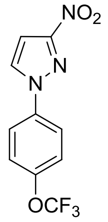

Transparent crystals (from diethyl ether), yield: 66%, mp 53–56 °C; ^{1}H NMR (400 MHz, DMSO): δ (ppm) = 7.37 (d, J^3=2.8 Hz, 1H, H$^{4\text{-Py}}$), 7.59-7.62 (m, 2H, H^{3,5}), 8.06-8.08 (m, 2H, H^{2,6}), 8.83 (d, J^3=2.8 Hz, 1H, H$^{5\text{-Py}}$); ^{13}C NMR (100 MHz, DMSO): δ (ppm) = 104.71 (s, C$^{4\text{-Py}}$), 119.96 (q, J^1=255.7 Hz, -OCF$_3$), 121.46 (s, C^{Ar}), 122.36 (s, C^{Ar}), 132.16 (s, C$^{5\text{-Py}}$), 137.25 (s, C^{Ar}), 147.66 (s, C^{Ar}) 156.67 (s, C$^{3\text{-Py}}$); EA calc. for $C_{10}H_6F_3N_3O_3$: C 43.97, H 2.21, N 15.38; found: C 44.18, H 2.33, N 15.30; ESIMS: *m*/*z*: 296.1 (M + Na)

Crystal data for compound 3k The crystal chosen for X-ray analysis was a clear colorless needle with the approximate dimensions of 0.6 × 0.3 × 0.3 mm. $C_{10}H_6F_3N_3O_3$ (273.18 g mol^{-1}) crystallizes in the monoclinic system, space group $P2_1$, with a = 4.2681 (9), b = 10.435 (2), c = 24.586 (5) Å, β = 90.20 (3), V = 1095.0 (4) Å^3, Z=4, μ(MoKα)= 0.157 mm^{-1}, and D_{calcd} = 1.657 cm^{-3}. A total of 2349 reflections were collected to $2\Theta_{max}$ = 50.13°(h: -5→0 k: -12→0, l: -29→29), of which 2042 were unique. In refinements, weights were used according to the scheme $w = 1/[\sigma^2(F_o^2)+(0.1084P)^2 +0.1267P]$, where $P = (F_o^2+2F_c^2)/3$. The refinement of 391 parameters (data-to-parameter ratio being 5.2) converged to the final agreement factors R = 0.0455 for 1454 reflections with Fo > 4σ (Fo) and R_w = 0.1494, and S = 1.008 for all observed reflections. The electron density of the largest difference peak was found to be 0.18 e Å^{-3}, while that of the largest difference hole was 0.25 e Å^{-3}.

3-Nitro-1-[3-(trifluoromethoxy)phenyl]-3-nitro-1*H*-pyrazole (3l)

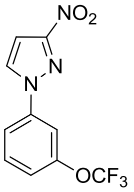

Peach solid, yield 64%, mp 32–34 °C; ^{1}H NMR (400 MHz, DMSO): δ (ppm) = 7.39 (d, J^3=2.8 Hz, 1H, $H^{4\text{-}Py}$), 7.49 (d, J^3=8 Hz, 1H, H^{Ar}), 7.75 (t, J^3=8 Hz, 1H, H^{Ar}), 7.97 (s, 1H, H^{Ar}), 8.02 (dd, J = 2 Hz, J^3=8 Hz 1H, H^{Ar}), 8.89 (d, J^3=2.8 Hz, 1H, $H^{5\text{-}Py}$); ^{13}C NMR (100 MHz, DMSO): δ (ppm) = 104.83 (s, $C^{4\text{-}Py}$), 112.38 (s, C^{Ar}), 118.34 (s, C^{Ar}), 119.95 (q, J^1=255.7 Hz, -OCF$_3$), 131.79 (s, C^{Ar}), 132.28 (s, $C^{5\text{-}Py}$), 139.67 (s, C^{Ar}), 148.94 (dd, J = 2.3 Hz, J = 3.8 Hz $C^{3\text{-}Ar}$) 156.69 (s, $C^{3\text{-}Py}$); EA calc. for $C_{10}H_6F_3N_3O_3$ C 43.97, H 2.21, N 15.38; found: C 44.08, H 2.31, N 15.45

1-(3-Nitrophenyl)-3-nitro-1*H*-pyrazole (3m)

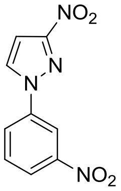

Pale peach solid, yield 62%, mp 121–122 °C; ^{1}H NMR (400 MHz, DMSO): δ (ppm) = 7.42 (d, J^3=2.8 Hz, 1H, $H^{4\text{-}Py}$), 7.90 (t, J^3=8.0 Hz, 1H, H^5), 8.31 (dd, J^3=8.0 Hz, J^4=2.2 Hz, 1H, H^6), 8.41 (dd, J^3=8.0 Hz, J^4=2.2 Hz, 1H, H^4), 8.72 (t, J^3=2.2 Hz, 1H, H^2); 9.01 (d, J^3=2.8 Hz, 1H, $H^{5\text{-}Py}$); ^{13}C NMR (100 MHz, DMSO) δ (ppm) = 105.06 ($C^{4\text{-}Py}$), 114.28, 122.81, 125.50, 131.47, 132.68, 139.08, 148.54, 156.87 ($C^{3\text{-}Py}$); EA calc. for $C_9H_6N_4O_4$: C 46.16, H 2.58, N 23.93; found: C 46.46, H 2.56, N 23.87

1-(4-Nitrophenyl)-3-nitro-1*H*-pyrazole (3n)

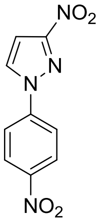

Light yellow solid, yield 65%, mp 165–167 °C; ^{1}H NMR (400 MHz, DMSO): δ (ppm) = 7.43 (d, J^3=2.8 Hz, 1H, $H^{4\text{-}Py}$), 8.22 (m, 2H, $H^{2,6}$), 8.44 (m, 2H, $H^{3,5}$), 8.98 (d, J^3=2.8 Hz, 1H, $H^{5\text{-}Py}$); ^{13}C NMR (100 MHz, DMSO): δ (ppm) = 105.32 ($C^{4\text{-}Py}$), 120.07, 125.42, 132.80 ($C^{5\text{-}Py}$),

142.63, 146.39, 157.25 ($C^{3\text{-}Py}$); EA calc. for $C_9H_6N_4O_4$: C 46.16, H 2.58, N 23.93; found: C 46.20, H 2.45, N 24.06

4-Nitro-1-phenylpyrazole (3o)

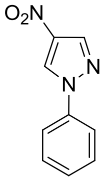

white solid, yield 53%, mp 128–129 °C (126–128 °C [6]); ^{1}H NMR (300 MHz, DMSO): δ (ppm) = 7.46 (t, J^3=7.8 Hz, 1H, H^4), 7.58 (t, J^3=7.8 Hz, 2H, $H^{3,5}$), 7.96 (d, J^3=8.1 Hz, 2H, $H^{2,6}$), 8.57 (s, 1H, $H^{3\text{-}Py}$), 9.66 (s, 1H, $H^{5\text{-}Py}$).

4-Nitro-1-phenylimidazole (3p)

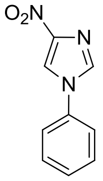

white solid, yield 75%, mp 187–188 °C (lit. 186–187 °C[7]); ^{1}H NMR (300 MHz, DMSO): δ (ppm) = 7.46 (t, J^3=7.5 Hz, 1H, H^4), 7.59 (t, J^3=7.5 Hz, 2H, $H^{3,5}$), 7.81 (d, J^3=8.1 Hz, 2H, $H^{2,6}$), 8.49 (s, J^3=1.2 Hz, 1H, H^{Im}), 9.01 (d, J^3=1.2 Hz 1H, H^{Im}).

3-Nitro-1-phenyl-1,2,4-triazole (3r)

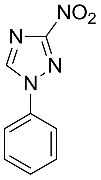

white solid, yield 50%, mp 135–136 °C (lit. 133 °C [5]); ^{1}H NMR (600 MHz, DMSO): δ (ppm) = 7.55-7.58 (tt, J^3 =7.8 Hz, J = 2.4 Hz, 1H, H^4), 7.63-7.66 (m, 2H, $H^{3,5}$), 7.92-7.94 (m, 2H, $H^{2,6}$), 9.59 (s, 1H, H^{Tri}); ESIMS: m/z: 213.1 (M + Na).

1-Phenyl-2-nitroimidazole (3s)

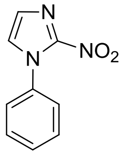

light yellow solid, yield 38%, mp 148–149 °C (lit. 141–142 °C (from methanol) [8]); H NMR (400 MHz, DMSO): δ (ppm) = 7.34 (d, J^3=1.2 Hz, 1H, H^{Im}), 7.54 (s, 5H, H^{Ar}), 7.74 (d, J = 1.2 Hz, 1H, H^{Im}); ^{13}C NMR (100 MHz, DMSO): δ (ppm) = 126.04, 128.40, 128.65, 129.38, 129.48, 137.11, 144.42 ($C^{2\text{-}Im}$); ESIMS: *m/z*: 190.2 (M + H).

References

1 Habraken, C. L.; Janssen, J. W. A. M. *J. Org. Chem.* **1971**, *36*, 3081-3084.

2 Jędrysiak, R. Synteza wybranych 1-podstawionych-4-nitroimidazoli w reakcjach transformacji pierścieni azolowych. Struktura i właściwości. Ph.D. Thesis, Silesian University of Technology, Gliwice, Poland, 2007 (in Polish).

3 Rusinov, V. L.; Myasnikov, A.V.; Chupakhin O. N.; Aleksandrov G.G *Chem. Heterocycl. Compd.* **1991**, *27,* 530-533.

4 Coburn, M. D. *J. Heterocycl. Chem.* **1970**, *7,* 455-456.

5 Chertkov, V. A.; Shestakova, A. K.; Davydov, D. V. *Khimiya Geterotsiklicheskikh Soedinenii*, 2011, *1*, 63–74; *Chem. Heterocycl. Compd*. 2011, *47*, 45-54.

6 Suwinski, J.; Swierczek, K.; Wagner, P.; Kubicki, M.; Borowiak, T.; Slowikowska, J. *J. Heterocycl. Chem*. **2003**, *40*, 523 – 528.

7 Salwińska, E.; Suwiński, J. *Pol. J. Chem* **1990**, *64,* 813-817.

8 Tertov, B. A.; Burykin, V. V.; Morkovnik A. S. Sposob polučeniâ 1-zamešennyh proizvodnyh 2-nitroimidazola. SU Patent 437763, July 30, 1974 (in Russian).
